# Using Eye Tracking to Explore Facebook Use and Associations with Facebook Addiction, Mental Well-being, and Personality

**DOI:** 10.3390/bs9020019

**Published:** 2019-02-18

**Authors:** Zaheer Hussain, Boban Simonovic, Edward J. N. Stupple, Maggie Austin

**Affiliations:** College of Life and Natural Sciences, School of Human Sciences, University of Derby, Derby DE22 1GB, UK; b.simonovic@derby.ac.uk (B.S.); e.j.n.stupple@derby.ac.uk (E.J.N.S.); m-austin@hotmail.co.uk (M.A.)

**Keywords:** Facebook addiction, personality, mental well-being, depression, anxiety, stress

## Abstract

Social networking sites (SNSs) have become ubiquitous in our everyday lives, and for all its communicative benefits, excessive SNS use has been associated with a range of negative health implications. In the present study, the authors use eye-tracking methodology to explore the relationship between individual differences in personality, mental well-being, SNS usage, and the focus of Facebook users’ visual attention. Participants (*n* = 69, mean age = 23.09, SD = 7.54) completed questionnaire measures for personality and to examine changes in depression, anxiety, stress, and self-esteem. They then engaged in a Facebook session while their eye movements and fixations were recorded. These fixations were coded as being directed to social and update areas of interest (AOI) of the Facebook interface. An exploratory analysis of personality factors revealed a negative correlation between openness to experience and inspection times for the updates AOI and an unexpected negative relationship between extraversion and inspection times for social AOI. There were correlations between changes in depression score and inspection of updates AOI, with reduced depression scores associated with increased inspection of updates. Finally, self-reported duration of participants’ typical Facebook sessions did not correlate with eye-tracking measures but were associated with increased Facebook addiction scores and greater increases in depression scores. These initial findings indicate that there are differences in the outcomes of interacting with Facebook which can vary based on Facebook addiction, personality variables, and the Facebook features that individuals interact with.

## 1. Introduction

Social networking sites (SNSs) such as Facebook, Instagram, and Twitter have become synonymous with daily-life activities with more than one billion daily users [1,2]. Social networking sites allow users to engage in a variety of activities including posting photos, communicating, updating profiles/status, checking the news, and playing games [3]. These activities can provide immediate gratification; however, long-term excessive SNS use can lead to negative health implications and even addiction to SNSs [4,5,6]. Several studies have linked online activities (such as online gaming, social networking site use, and online gambling) to substance use disorders [7,8], and engaging in excessive use of SNSs can potentially lead to addiction [9]. Social networking site addiction has been defined as the inability to regulate SNS usage which then leads to negative personal outcomes [10]. Social networking site addicts have been found to experience emotional, relational, and health-related problems [11]. Social networking site addiction prevalence rates vary across countries; 1.6% was reported among a Nigerian sample [12], 8.6% was reported among a Peruvian sample [13], and 12% was reported among a Chinese sample [14].

Researchers studying addictive SNS use have revealed findings that show different aspects of addictive use. Research by Kuss, Griffiths, Karila, and Billieux [15] showed that SNS users who are not in a romantic relationship are more prone to developing SNS addiction than users who have partners. Research [16] has reported that younger SNS users tend to score higher on SNS addiction scales than older users. Other research [17] has reported SNS addiction in older users, in males [18] and females [19,20]. When considering gender, research has reported higher levels of addictive SNS use among men [18]. Whilst some studies have reported that addictive SNS use is more prevalent among females [21,22]. On the contrary, there is evidence showing that SNS addiction is unrelated to age [23] and gender [14,24]. 

Studies have provided interesting insights into the impact that addictive SNS use can have on health and well-being. Facebook addiction has been linked to relationship dissatisfaction [25], subjective happiness, and subjective vitality [26]. Research studies have reported associations between SNS dependence and poor sleep quality [27]. Blachnio, Przepiorka, and Pantic [28] found that Facebook addiction was associated with low self-esteem and negatively associated with life satisfaction. Research studies [29,30] have also reported significant associations between Facebook addiction and increased time spent on Facebook. Together, these research findings highlight several factors that are associated with addictive SNS use.

The fact that depression often involves social withdrawal [31], high levels of depression symptoms have been linked to problematic SNS use [23,29,32,33], pathological Internet use [34], and Internet addiction [35,36]. Research by Morrison and Gore [37] found that there was a close relationship between Internet addiction tendencies and depression, such that Internet addicted respondents were more depressed. Similarly, Huang et al. [38] reported that 9% of the participants in their study were classified as problematic Internet users and 25.5% of the participants with depression developed problematic Internet use (PIU). Lin, Ko, and Wu [39] reported that depressive symptoms were positively correlated with Internet addiction. On the contrary, Andreassen et al. [19] reported that depression contributed negatively to SNS addiction. Similarly, Shensa, et al. [40] found that problematic SNS use was strongly associated with increased depressive symptoms in US young adults. Taken together, these studies suggest that there may be associations between depression symptoms and SNS addiction.

Research studies have reported links between SNS addiction and anxiety [29,41]. Koc and Gulyagci [23] reported that one of the predictors of Facebook addiction was anxiety. Andreassen et al. [19] found a positive correlation between anxiety and addictive SNS use in their large-scale study. Research has also shown links between pathological Internet use/Internet addiction and anxiety [34,35]. Research by Weinstein et al. [42] revealed a correlation between Internet addiction and social anxiety. Interestingly, the researchers did not find a preference for SNSs among socially anxious participants. Altogether, most studies have shown that anxiety appears to be correlated with SNS use and Internet addiction. Anxious people may find it difficult to communicate face-to-face, so online communication is then preferred over face-to-face communication [19].

The role of personality traits in understanding SNS use has been investigated by several studies. For instance, research studies [16,20] have shown that neuroticism is positively related to SNS addiction. Extraversion has been shown to be positively correlated with SNS addiction [16,43]. Wang, Ho, Chan, and Tse [44] reported that SNS addiction was predicted by high extraversion. Low levels of conscientiousness have been associated with SNS addiction [20,43]. Research [15] has reported that the personality characteristics of neuroticism and impulsivity may put individuals at risk for developing Internet addiction. The trait of narcissism has been found to be positively associated with social networking activities [45,46], with evidence showing that Facebook addiction is positively correlated with narcissism [47]. These studies show interesting associations between personality traits and SNS addiction. Some research has reported a negative relationship between self-esteem and addictive use of SNSs [29,43,47,48]. Research by Bányai et al. [32] found that the at-risk SNS users reported low levels of self-esteem. Social networking site addiction has been empirically linked to low self-esteem in several studies [29,43,49]. There are also a few studies [50,51,52,53] that have reported significant and positive associations between problematic SNS use and stress. Taken together, previous studies investigating problematic SNS use have reported associations with personality and mental health. Survey methods have been predominately used in these studies, researchers [6] have recently called for the use of new and innovative methods to examine SNS use. 

Eye tracking is a new and original method of research; this method has been used to investigate areas of interest (AOI), gaze, and general eye movements. Only a few studies have used this method in the context of SNS use. For instance, Seidman and Miller [54] examined participants’ gaze while observing Facebook profiles of strangers varying in gender and physical attractiveness. Results showed that participants paid more attention to the physical appearance (main profile photograph) of female than of male profile owners and to the personal information (likes and interests) of male than of female profile owners. Vraga, Bode, and Troller-Renfree [55] used eye tracking to gauge attention to content in Facebook. The results revealed that news and social content garnered the most amount of attention. The results also showed that richer content (e.g., pictures, links) enhanced attention. Other eye-tracking studies have investigated virtual navigation [56], immersive environments [57], visual observations of shoppers viewing products [58,59], webpage usability [60], attentional bias of smokers towards smoking-related and aversive cues [61], source evaluations during web searches [62], eye movements in an online shopping context [63], and eye fixations on tourism websites [64]. These studies show the capabilities of eye-tracking and how the method can be used to yield interesting insights.

Given the unprecedented popularity of Facebook among Internet users across the world there may be unique aspects associated with the development of addiction with this SNS [11]. Previous research has shown that problematic SNS use can result in negative consequences [6]. Research studies appear to suggest that individuals with anxiety or depression are motivated to use SNSs to find relief or social support [11]. More recently, research [65] has shown that increased SNS use is associated with poor sleep, anxiety, depression, and low self-esteem. It is important to examine the potential negative consequences of SNS use and the impact on health and well-being. The aim of the present study was to explore the relationship between individual differences in personality, mental well-being, SNS usage, and the focus of Facebook users’ visual attention. This method focused on points of interest among participants when using Facebook in a laboratory setting. Changes in psychological states after a short Facebook session was also investigated, this made the study unique as previous studies have not done this. The present study also explored the relationships between depression, anxiety, stress, self-esteem, and Facebook addiction. 

## 2. Methods

### 2.1. Design

A correlational design was used to explore the relationships between Facebook usage, personality, and mental well-being. Further relationships between Facebook usage measures and changes in mental well-being were also explored. The variables included Facebook Addiction; Facebook Account length; Personality measures: Extraversion, Openness, Agreeableness, Conscientiousness, Emotional Stability; Eye-tracking measures: Social Facebook Total Inspection-time (seconds), Facebook Updates Total Inspection-time (seconds); Facebook Session Length (a self-reported indication of the duration of a typical Facebook session); mental well-being scores: Depression, Anxiety, Stress, Self Esteem, as well as change scores for these variables after the Facebook session: Depression change score, Anxiety change score, Stress change score and Self Esteem change score.

### 2.2. Participants

The sample consisted of 69 participants aged over 18 years, with a mean age of 23.09 (SD = 7.54). There were 47 (68.1%) female and 22 (31.9%) male participants. Most participants (98.6%, *n* = 68) were students at a large university in the UK, one participant was in employment. The participants stated that their ethnicity was White (78.3%, *n* = 54), Asian (8.7%, *n* = 6), Black (4.3%, *n* = 3), African (4.3%, *n* = 3), and Other, Mixed/Multiple ethnic group (4.3%, (*n* = 3). Participants stated that they were single (59.4%, *n* = 41), in an intimate relationship (34.8%, *n* = 24), or married (5.8%, *n* = 4).

### 2.3. Materials 

Data were collected with the use of *Qualtrics* online survey software, two surveys were developed (survey 1 for completion before Facebook use, survey 2 for completion after Facebook use). The surveys were comprised of four psychological measurement instruments (presented in English) that together assessed the associations between Facebook addiction, self-esteem, mental well-being, and personality. The four measurement instruments were as follows.

#### 2.3.1. The Bergen Facebook Addiction Scale

The Bergen Facebook Addiction Scale (BFAS; [16]) was used to measure addiction to Facebook. The scale is anchored in general addiction theory and operationalizes Facebook addiction according to six basic addiction symptoms (i.e., salience, conflict, mood modification, withdrawal, tolerance, and relapse) [66]. All questions concern experiences occurring over the past year and are rated on a 5-point Likert scale spanning from Very rarely (1) to Very often (5) (e.g., “How often during the last year have you become restless or troubled if you have been prohibited from using Facebook?”). The items correspond with diagnostic addiction criteria [67]. Overall scores can be obtained by summing the responses (ranging from 6 to 30 points) with higher scores indicating a higher level of addictive Facebook use. The internal consistency and reliability of the BFAS has been reported to be good (α = 0.83; [16]).

#### 2.3.2. Rosenberg Self-Esteem Scale

The Rosenberg Self-Esteem Scale (RSES; [68]) was used to assess participants’ levels of self-esteem. The RSES is a 10-item scale, all statements are rated on a 4-point Likert scale ranging from Strongly agree (0) to Strongly disagree (3). The scale measures both positive and negative feelings about the self (example questions include, “All in all, I am inclined to feel that I am a failure” and “I am able to do things as well as most other people”). Scores are summed with higher scores indicating higher levels of self-esteem. The internal consistency of the RSES has been reported to be good (α = 0.91; [69]). 

#### 2.3.3. The 21-Item Short form Depression, Anxiety, and Stress Scale

The 21-item short form Depression, Anxiety, and Stress Scale (DASS-21; [70]) was used to assess symptoms of depression, anxiety, and stress. The scale comprises three 7-item sub-scales covering the three symptoms that are rated on a 4-point Likert scale ranging from Never (0) to Almost Always (3). Example questions are as follows; “I found it hard to wind down”, “I felt down-hearted and blue”, and “I found myself getting agitated”. Scores are summed and then multiplied by two (to make scores comparable to the DASS-42), overall scores ranged from 0–42 with high scores indicating elevated depression, anxiety, and stress. The internal consistency of the DASS-21 has been reported to be highly reliable (depression α = 0.94, anxiety α = 0.87, stress α = 0.91; [71]).

#### 2.3.4. The Ten Item Personality Inventory

Personality traits were assessed using the Ten Item Personality Inventory (TIPI; [72]), the TIPI is a valid measure of the Big Five (Five-Factor Model) personality dimensions. The TIPI comprises ten items using a 7-point rating scale (1 = Disagree strongly to 7 = Agree strongly) and five sub-scales; Extraversion, Agreeableness, Conscientiousness, Emotional stability, Openness. Gosling et al. [72] report that the TIPI has adequate levels in terms of: (a) convergence of widely used Big-Five measures in self, observer, and peer reports, (b) test–retest reliability, (c) patterns of predicted external correlates, and (d) convergence between self and observer ratings. 

#### 2.3.5. Eye Tracking Measurements

Eye movements were recorded with the eye-gaze binocular system Tobii-X2-30, with a remote binocular sampling rate of 30 Hz and an accuracy of about 0.45°. The X2 Eye Tracker is a stand-alone eye tracker, and it was attached to a laptop (Dell, Precision M6700, 2.70 Ghz). Participants were seated approximately 70 cm from the laptop monitor. The Tobii measured 184 mm (7.2’’) in length and enabled tracking at close distances (up to 36° gaze angle). Fixations were identified using a fixation radius of 20 pixels and a minimum fixation duration of 100 ms or above. Before engaging with Facebook activity, a 9-point calibration routine was executed. Each data point was identified with a timestamp and “X, Y” coordinates, these coordinates were processed further into fixations and overlaid on a Facebook page. Basic eye-tracking parameters such as inspection time and coordinates were recorded. To avoid methodological artefacts, eye tracking metrics were delineated through fixation filters. Hence, six AOI were obtained with the size of 236 × 212 pixels (Chat); 873 × 442 pixels (Newsfeed); 37 × 328 pixels (Notifications); 482 × 294 pixels (Trending—advertisement); 288 × 214 pixels (Trending 2—news sports); and 754 × 201 pixels (Who’s online). For each participant, the inspection time within each AOI was calculated.

### 2.4. Procedure

Following consent, participants completed survey 1 which consisted of basic demographic questions (i.e., age, gender, social status) Facebook use questions (i.e., How long have you had a Facebook account? How long (in minutes) does a Facebook session last?), the DASS-21, BFAS, TIPI, and RSES. Upon completion of Survey 1, participants engaged in Facebook activity for 3 minutes and eye-tracking measurements were taken during this period. Thereafter, participants completed survey 2 which included the DASS-21 and the RSES. Finally, participants were debriefed and thanked for their participation. Students received participation credits and were entered into a prize draw to win book vouchers.

### 2.5. Ethics

The study was carried out in accordance with the Declaration of Helsinki (i.e., adhering to the ethical principles for research involving human participants) and adhering to the British Psychological Society ethical guidelines. The university’s ethics committee approved the study, all participants were informed about the study and all provided informed consent.

## 3. Analytic Strategy

Descriptive statistics in terms of the means and standard deviations (SDs) were calculated. Due to violations of parametric assumptions in the data non-parametric Spearman’s Rho correlation coefficients were calculated to assess the inter-relationships between study variables. 

## 4. Results

### 4.1. Descriptive Statistical Analysis

Descriptive statistical analyses were performed on the study variables (see Table 1). The observed levels of Facebook addiction (mean = 12.56, SD = 4.14) were low. In relation to the other main study variables; self-esteem scores (mean = 20.10, SD = 4.43) were moderate, depression scores (mean = 10.31, SD = 7.41) were low, anxiety scores (mean = 9.30, SD = 7.27) were low, stress scores (mean = 15.40, SD = 6.49) were low. Participants spent almost 2 min viewing the updates areas of their Facebook page (Facebook updates total inspection time, mean = 118.07, SD = 44.12).

### 4.2. Eye Tracking Analyses

Facebook use and personality were explored by correlating the big five personality scores, the DASS scores, Facebook addiction scores with the length of the Facebook account, session length, inspection times of Social, and update AOI to examine whether there were any trends associating individual differences in personality and mental health variables and Facebook interaction. These correlations are presented in Table 2. To reduce Type 1 error, the threshold alpha level was set at *p* < 0.01 for all analyses. There were significant positive relationships between Facebook addiction and session length. There was also a negative correlation between Openness to experience and inspection times for the Newsfeed/Update AOI.

To explore the relationship between patterns of Facebook use and changes in mental state, Spearman’s Rho correlations were conducted between areas of interest, and changes in the sub-scales of the DASS, and self-esteem (see Table 3 for details). Areas of interest were sorted into two categories: social features (chat and who is online) and updates (newsfeed, notifications, trending), and the total fixation duration was calculated for these areas and these times were correlated with depression, anxiety, stress, and self-esteem change scores. The change scores for the DASS and self-esteem variables were calculated by subtracting the post-Facebook viewing score from the pre-Facebook viewing score for each of the subscales. A significant negative correlation was found between depression change score and Inspection time for the Updates AOI, such that increased viewing of updates was associated with lower depression scores.

## 5. Discussion

The aim of the present study was to investigate personality and individual differences associated with Facebook use by using eye-tracking as a direct measure of the focus of attention. The results showed that Facebook addiction was positively associated with session length suggesting that increased time spent using Facebook was associated with increased risk of experiencing problematic use of Facebook. This finding is similar to previous research (e.g., [29,30]). A negative association was found between openness to experience and inspection times for the Newsfeed/Update AOI. This finding is similar to previous research [73] and it suggests that as people become less open to experiences, their time spent viewing the newsfeed area of Facebook increases. They may spend more time observing information and updates of others on Facebook rather than engage in social activities. This could lead to loneliness, low self-esteem, and low life satisfaction. These negative psychological traits have been found to be associated with Facebook addiction [28]. There is an alternative interpretation of the negative correlation between openness to experience and newsfeed engagement, which is that the newsfeed is most attractive to the more closed-minded user. This view is supported by Bessi et al. [74] who have shown that social media users typically engage with information that conforms to their beliefs. A content analysis of Facebook newsfeed algorithms showed newsfeed content was determined by factors including “explicitly expressed user interests”, “prior user engagement”, “implicitly expressed user preferences”, and “negatively expressed preferences” [75]. In combination, these studies would predict that we all tend to prefer SNS content that confirms our prejudices; however, our eye-tracking data suggest the lowest scorers in openness to experience may be particularly attentive to such self-confirmatory messages. 

Participants stated that their Facebook session lengths tended to last 105 minutes (almost 2 hours per session), this suggests that SNSs can be very engaging and time consuming. Interestingly, viewing/inspecting Facebook updates was associated with a decrease in depression change scores. This finding partially supports previous research by Lee, Cheung, and Thadani [76]; the participants may be using Facebook to regulate their mood which could lead to problematic Facebook use. In contrast to this finding, it was found that increasing Facebook session lengths was associated with increases in depression change scores. This supports previous research findings (e.g., [29,40,65]) showing problematic SNS use or increased use to be associated with an increase in depressive symptoms. It is worth noting, however, that the study entailed a short period of Facebook use and participants who preferred longer sessions may have experienced the brevity of the task negatively. Finally, it is worth noting that the study findings revealed a near significant result showing an increase in Facebook session length was associated with an increase in anxiety scores. This was consistent with previous findings [65]; however, further research with increased sample sizes would be required to understand whether this effect is reliable and what might cause an increase in anxiety associated with SNS use.

The present research findings reveal interesting insights into Facebook use with associations being found between several psychiatric disorder symptoms and problematic SNS use. When observing change scores; depression, anxiety, and stress scores decreased after the three-minute Facebook session, while self-esteem increased. Facebook users may be motivated to use the SNS to find relief [11]. Furthermore, it can be speculated that Facebook users may be using the SNS to regulate mood, this has been found in previous research [77] among Facebook addicts. Hormes et al. [77] reported that Facebook addicts found it difficult to regulate their emotions and were susceptible to both substance and non-substance addiction. The present study findings may help in developing intervention programs to treat the symptoms of depression, anxiety, and stress. One possible intervention could be the use of a mindfulness smartphone application to help decrease technology use and improve wellbeing, the first author is currently investigating this.

Several limitations of the study need to be discussed. Due to the cross-sectional nature of the study design, it is not possible to discern the direction of causality. There is the possibility that recorded eye tracking behavior may have been influenced by feelings of being observed. However, the use of new and innovative methods, as well as the use of validated measurement instruments, represents one of the key strengths of the present study. Eye tracking methods represent an important starting point to illustrate advantages of innovative methods in psychology. Future research could use eye tracking to monitor activities in new SNSs and in clinical samples. It is important to note that self-reported data was and will always be an important source when dealing with maladaptive behavior of an individual [78] but also has well-known limitations. Combining self-reported data with behavioral tracking data may, however, increase the validity of future study findings [78]. Furthermore, future research must address the presence of specific addiction symptoms among users and go beyond negative consequences of SNS use, and it appears necessary to conduct further psychophysiological studies [22]. Using longitudinal study designs and real-time smartphone data, also known as psychoinformatics [78,79], would resolve some of the aforementioned limitations. The relationship shown between openness to experience and newsfeed engagement also warrants further exploration with regards to individual differences in SNS engagement and potential susceptibility to belief polarization. Using innovative methods to investigate psychological phenomena will lead to new opportunities for scientific insights. The findings could help with the development of interventions targeting the main variables in this study and with the aim of preventing negative consequences. Eye tracking methods provide new ways to assess maladaptive behaviors and may be used to inform treatment.

## Figures and Tables

**Table 1:** Means (standard deviations) for study variables.

Study Variables	Mean (SD)
Facebook Addiction	12.56 (4.14)
Facebook Account length (months)	70.0 (31.12)
Extraversion	4.59 (1.52)
Openness	5.56 (1.06)
Agreeableness	4.18 (1.04)
Conscientiousness	5.33 (1.20)
Emotional Stability	4.54 (1.40)
Social Facebook Total Inspection Time (seconds)	30.32 (45.18)
Facebook Updates Total Inspection Time (seconds)	118.07 (44.12)
Facebook Session Length (minutes)	105.22 (96.47)
Depression	10.31 (7.41)
Anxiety	9.30 (7.27)
Stress	15.40 (6.49)
Self-Esteem	20.10 (4.43)
Depression Change Score	−2.70 (4.55)
Anxiety Change Score	−1.37 (5.52)
Stress Change Score	−2.89 (4.19)
Self-Esteem Change Score	0.61 (2.06)

**Table 2:** Personality and individual differences measures and inspection time correlations.

	Account Duration	Facebook Session Length	Social Feature Inspection	Update/ Newsfeed Inspection	Facebook Addiction
Facebook Addiction	*r_s_* = 0.163 *p* = 0.216	*r_s_* = 0.366 * p = 0.004	*r_s_* = 0.035 *p* = 0.800	*r_s_* = −0.091 *p* = 0.507	
Extraversion	*r_s_* = −0.024 *p* = 0.856	*r_s_* = 0.271 *p* = 0.038	*r_s_* = −0.322 *p* = 0.016	*r_s_* = −0.119 *p* = 0.387	*r_s_* = 0.146 *p* = 0.253
Openness	*r_s_* = 0.125 *p* = 0.345	*r_s_* = 0.264 *p* = 0.044	*r_s_* = 0.052 *p* = 0.708	*r_s_* = −0.353 * p = 0.008	*r_s_* = 0.133 *p* = 0.300
Agreeableness	*r_s_* = −0.211 *p* = 0.109	*r_s_* = 0.044 *p* = 0.740	*r_s_* = −0.204 *p* = 0.135	*r_s_* = −0.042 *p* = 0.759	*r_s_* = 0.080 *p* = 0.534
Conscientiousness	*r_s_* = 0.085 *p* = 0.524	*r_s_* = −0.113 *p* = 0.395	*r_s_* = −0.151 *p* = 0.272	*r_s_* = 0.167 *p* = 0.222	*r_s_* = −0.063 *p* = 0.625
Emotional Stability	*r_s_* = −0.039 *p* = 0.768	*r_s_* = −0.038 *p* = 0.778	*r_s_* = 0.027 *p* = 0.844	*r_s_* = 0.032 *p* = 0.814	*r_s_* = −0.184 *p* = 0.814
Depression	*r_s_* = 0.353 * p = 0.005	*r_s_* = −0.008 *p* = 0.950	*r_s_* = 0.330 *p* = 0.012	*r_s_* = 0.080 *p* = 0.553	*r_s_* = 0.241 *p* = 0.057
Anxiety	*r_s_* = −0.123 *p* = 0.345	*r_s_* = −0.076 *p* = 0.561	*r_s_* = −0.121 *p* = 0.371	*r_s_* = 0.055 *p* = 0.686	*r_s_* = 0.161 *p* = 0.208
Stress	*r_s_* = 0.132 *p* = 0.312	*r_s_* = −0.086 *p* = 0.511	*r_s_* = 0.011 *p* = 0.937	*r_s_* = 0.095 *p* = 0.483	*r_s_* = 0.039 *p* = 0.761
Self Esteem	*r_s_* = −0.096 *p* = 0.468	*r_s_* = 0.108 *p* = 0.415	*r_s_* = −0.121 *p* = 0.379	*r_s_* = −0.069 *p* = 0.617	*r_s_* = −0.120 *p* = 0.350

Note: Effects significant at *p* < 0.01 are denoted with *.

**Table 3:** Correlations between areas of interest, session length, and changes in depression, anxiety, and stress scores.

	Social Facebook Total Inspection-time	Facebook Updates Total Inspection-time	Facebook Session Length
Social Facebook Total Inspection Time	1.000		
Facebook Updates Total Inspection Time	*r_s_* = −0.096, *p* = 0.456	1.000	
Facebook Session Length	*r_s_* = −0.021, *p* = 0.875	*r_s_* = 0.026, *p* = 0.847	1.000
Depression Change Score	*r_s_* = − 0.173, *p* = 0.197	*r_s_* = −0.365, *p* = 0.005 *	*r_s_* = 0.293, *p* = 0.022
Anxiety Change Score	*r_s_* = 0.021, *p* = 0.877	*r_s_* = −0.200, *p* = 0.136	*r_s_* = 0.248, *p* = 0.054
Stress Change Score	*r_s_* = −0.226, *p* = 0.092	*r_s_* = −0.098, *p* = 0.469	*r_s_* = 0.187, *p* = 0.148
Self-Esteem Change Score	*r_s_* = 0.020, *p* = 0.884	*r_s_* = 0.042, *p* = 0.762	*r_s_* = −0.111, *p* = 0.403

Note: Effects significant at *p* < 0.01 are denoted with *.
